# Always-On Sub-Microwatt Spiking Neural Network Based on Spike-Driven Clock- and Power-Gating for an Ultra-Low-Power Intelligent Device

**DOI:** 10.3389/fnins.2021.684113

**Published:** 2021-07-20

**Authors:** Pavan Kumar Chundi, Dewei Wang, Sung Justin Kim, Minhao Yang, Joao Pedro Cerqueira, Joonsung Kang, Seungchul Jung, Sangjoon Kim, Mingoo Seok

**Affiliations:** ^1^Department of Electrical Engineering, Columbia University, New York City, NY, United States; ^2^Samsung Electronics, Seoul, South Korea

**Keywords:** always-on device, spiking neural network, event-driven architecture, neuromorphic hardware, clock and power gating

## Abstract

This paper presents a novel spiking neural network (SNN) classifier architecture for enabling always-on artificial intelligent (AI) functions, such as keyword spotting (KWS) and visual wake-up, in ultra-low-power internet-of-things (IoT) devices. Such always-on hardware tends to dominate the power efficiency of an IoT device and therefore it is paramount to minimize its power dissipation. A key observation is that the input signal to always-on hardware is typically sparse in time. This is a great opportunity that a SNN classifier can leverage because the switching activity and the power consumption of SNN hardware can scale with spike rate. To leverage this scalability, the proposed SNN classifier architecture employs event-driven architecture, especially fine-grained clock generation and gating and fine-grained power gating, to obtain very low static power dissipation. The prototype is fabricated in 65 nm CMOS and occupies an area of 1.99 mm^2^. At 0.52 V supply voltage, it consumes 75 nW at no input activity and less than 300 nW at 100% input activity. It still maintains competitive inference accuracy for KWS and other always-on classification workloads. The prototype achieved a power consumption reduction of over three orders of magnitude compared to the state-of-the-art for SNN hardware and of about 2.3X compared to the state-of-the-art KWS hardware.

## Introduction

An spiking neural network (SNN) classifier is an attractive option for ultra-low-power intelligent internet-of-things (IoT) devices. It is promising especially for always-on functions due to their spike-based operation for computation and communication, allowing their switching activity and power to scale smoothly with the input activity rate. An SNN, therefore, is suitable for applications like keyword spotting (KWS) or face recognition in surveillance, thanks to its event-driven operation.

Spiking neural network based hardware work so far, however, focused on either the acceleration of neural simulations or the improvement of both performance and energy efficiency. In other words, they are not designed for always-on function. For example, Neurogrid (Benjamin et al., 2014) targets large-scale neural simulations. It employs analog neurons and address event representation (AER) for communication, the latter using a multi-bit bus. SpiNNaker (Painkras et al., 2013) also targets neural simulation and employs an array of embedded digital processors communicating asynchronously. Yang et al. presented multiple works that targeted large scale neural simulations. In CerebelluMorphic (Yang et al., 2021b) they simulated portions of the cerebellum related to motor learning using 6 field programmable gate array (FPGA) chips that communicate using a multicast router. In BiCoSS (Yang et al., 2021c) they presented a platform with 35 FPGA chips connected to realize real-time computation of biological activities in multiple brain areas. In another work (Yang et al., 2021d), they presented an event-based processing algorithm that used piecewise linear approximation and binarization for efficient implementation of credit assignment to neurons in neuromorphic hardware. On the other hand, TrueNorth (Akopyan et al., 2015) was designed to be a scalable low power neurosynaptic inference engine for SNNs. The architecture was event-driven and employed synchronous circuits for computation blocks and asynchronous circuits for communication. Also, Tianjic chip was designed to support inference only with both neuromorphic and deep-learning models (Pei et al., 2019). Some works proposed architectures for both the training and inference of SNNs. Koo et al. (2020) introduced the implementation of a stochastic bit and used it in the realization of a neuron and synapse. They support on-chip training and inference with the synapse being stochastic in training and neuron being stochastic in both training and inference. Chen et al. (2018) presented an SNN accelerator with on-chip spike-timing-dependent plasticity (STDP) based learning. This chip has 64 cores that communicate using a network-on-chip (NoC) with each core supporting 64 leaky integrate and fire (LIF) neurons. Also, Loihi (Davies et al., 2018) was designed to support a variation of the current based dynamics LIF neuron model and a wide range of synaptic learning rules for both supervised and unsupervised learning. It is built for performance. It has 128 cores, three x86 cores, off-chip interfaces and an asynchronous NoC for communication between cores. Also, Seo et al. (2011), implemented a scalable architecture with a set of 256 neurons and transposable memory for synapses in near-threshold voltage (NTV) circuits. It mapped an auto-associative memory model. Some other works implemented different learning rules for on-chip training. Knag et al. (2015), implemented a feature extractor based on a sparse coding algorithm using LIF neurons. Park et al. (2019), developed a new neuromorphic training algorithm and hardware which supports low overhead on-chip learning. Some of these chips e.g., (Akopyan et al., 2015; Davies et al., 2018) employ asynchronous logic such as quasi-delay-insensitive (QDI) dual-rail dynamic logic or bundled data communication. Asynchronous logic circuits are, however, generally bulkier and power-hungrier than the single-rail static counterpart and also not very voltage-scalable (Chen et al., 2013; Liu et al., 2013) and bundled data communication incurs significant overhead because of the handshake. Some other chips employ power-efficient static logic (Chen et al., 2018; Davies et al., 2018; Park et al., 2019; Pei et al., 2019), but they target high throughput, not always-on function. As a result, they exhibit a power consumption of more than tens of mW, which makes it difficult to use them for always-on functions.

In this work, we focus on ultra-low-power *always-on* inference hardware and propose an SNN classifier consuming less than 300 nW. Our architecture uses fully spike-based event-driven operation and only static logic operating at a NTV to achieve such low power. Specifically, our design is centered around the neurosynaptic core. It is implemented using static gates and spike-driven (i) spatiotemporally fine-grained clock generation, (ii) clock-gating, and (iii) power-gating. Also, the communication between neurosynaptic cores is free from information loss due to the collision of spikes, despite using only wires to connect the cores. The architecture exhibits active power consumption that is proportional to the input rate due to its event-driven nature.

We also employ the technique in Cao et al. (2014) to train a neural network with binary weights and use the weights for the SNN we intend to deploy. The use of binary weights is a recent development in deep learning for making inference efficient (Courbariaux et al., 2015). They are of special interest because of their reduced memory footprint and simple computations. They are well suited for low power hardware and attain close to state of the art accuracy on datasets like MNIST. On the other hand, we keep the activations as spike-rate-coded multi-bit values, which improves the model’s inference accuracy.

We prototyped an SNN classifier in 65-nm LP CMOS technology. It has 5-layers and a total of 650 neurons and 67,000 synapses. It consumes 2.3–6.8X lower power at state-of-the-art accuracies on two well-known KWS benchmarks, i.e., Google Speech Command Dataset (GSCD) for multi-keyword recognition (Warden, 2018) and HeySnips for single-keyword spotting (Coucke et al., 2019).

In the remaining portion of this manuscript, we will present our SNN hardware architecture and the experimental results. In section “Materials and Methods,” we discuss the high-level SNN classifier architecture, elaborate on each of the components of the neurosynaptic core and introduce the experiment setup. In section “Results,” we present the results and finally conclude in section “Discussion.”

## Materials and Methods

**FIGURE 1:**
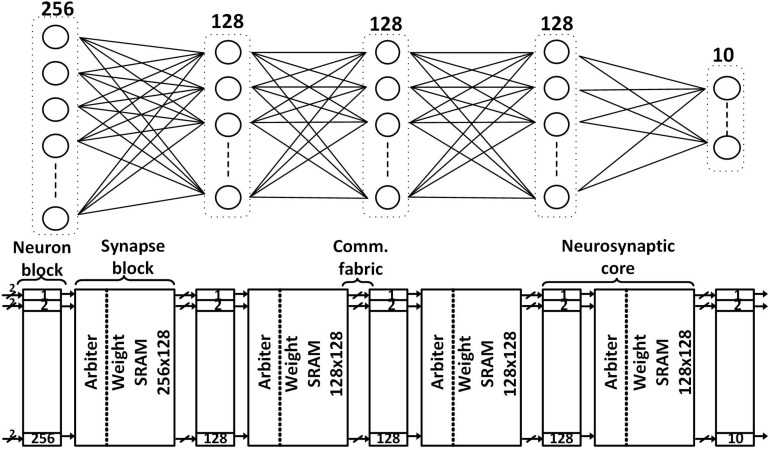
The proposed SNN classifier architecture **(bottom)** with the maximum supported network size **(top)** for always-on functions like keyword spotting.

The SNN classifier in our proposed design is depicted in Figure 1. It can support a fully connected network as large as 256-128-128-128-10 with binary weights onto five neurosynaptic cores which is sufficient to support the KWS task. We map each layer of the network to a different neurosynaptic core. The neuron block in the neurosynaptic core for the input layer has 256 neurons while the ones for the hidden layers, each contains 128 neurons. Each neuron has its own hardware and thus they can process in parallel. The size of each layer can be altered to make it smaller by configuring the neurosynaptic core using the scan chain. The architecture needs to change if a much larger network needs to be supported while not increasing the area for tasks like object identification in a security video, necessitating time-sharing of neuron hardware.

The input and hidden neurosynaptic cores have a neuron block and a synapse block while the output neurosynaptic core has only a neuron block. A neuron block contains all the IF neurons in that layer, a synapse block has (i) an arbiter, (ii) an SRAM storing up to 256-by-128 binary weights for the input neurosynaptic core and up to 128-by-128 binary weights for the hidden neurosynaptic cores, and (iii) a spike generator that simultaneously generates 128 spikes.

### Neuron Block

**FIGURE 2:**
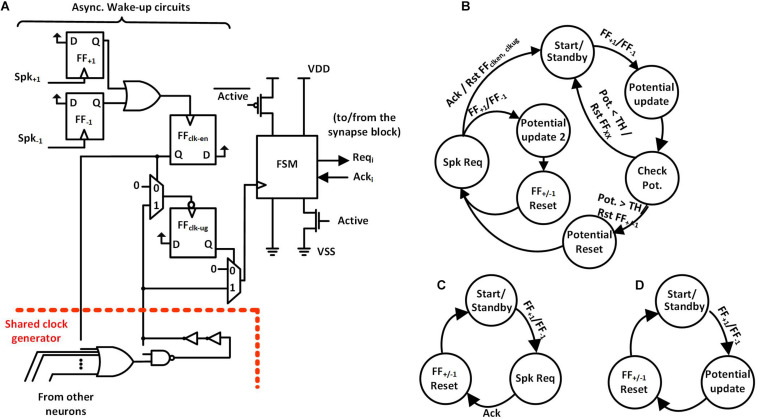
**(A)** Neuron Block Architecture with wake-up circuits on the left, FSM with zigzag power gating on the right and Shared Clock generator on the bottom. **(B)** Hidden Neuron FSM. **(C)** Input Neuron FSM. **(D)** Output Neuron FSM.

We propose a spike-event-driven architecture. Figure 2 shows the neuron block based on that architecture. Each neuron has (i) asynchronous wake-up circuits and (ii) a synchronous finite state machine (FSM). Also, all the neurons in a neuron block share a clock generator based on a ring oscillator. The architecture contains fine-grained clock-generation and clock-gating circuits based on spike input as an event. In the absence of input spikes, each neuron gates its clock and also power-gates the non-retentive parts of the neuron using zigzag power-gating switches (PGSs) (Cerqueira and Seok, 2017), to reduce static power dissipation. In zigzag power-gating, if the circuit in the power down state, the gates are left in alternate states by default, reducing the capacitance that needs to be charged while transitioning into power on state.

**FIGURE 3:**
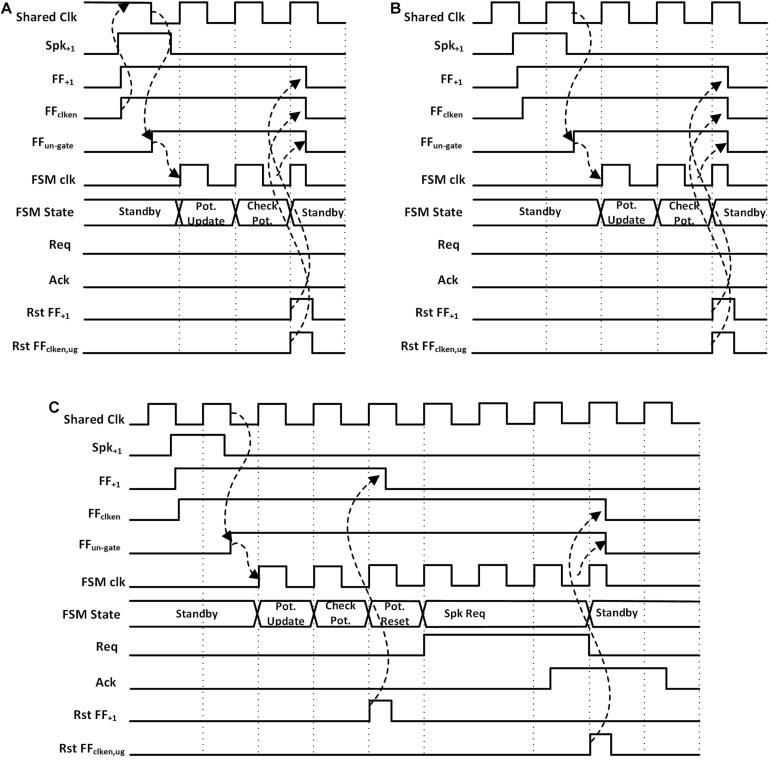
Waveforms for a hidden neuron FSM when **(A)** potential is less than the threshold and shared clock was disabled, **(B)** potential is less than the threshold and shared clock is running (assume other neurons in the same neuron block are active), **(C)** potential is greater than the threshold and shared clock is running.

The wake-up circuit of each neuron (Figure 2A, left) has the static flip-flops, FF_+__1_ and FF_–__1_, which detect the rising edge of the incoming spikes from two inputs, Spk_+__1_ and Spk_–__1_. Positive spikes which increase the potential of the neuron are directed to Spk_+__1_ and negative spikes which decrease the potential to Spk_–__1_. As shown in Figure 3A, the detection of a spike makes the output of the clock-enable flip-flop (FF_clk–en_) high. It also un-gates the PGS of the neuron. Thanks to the zigzag PGS, the ungating (i.e., wake-up) is done in a single clock cycle.

This process starts up the shared clock generator in the neuron block if it was not already started by another neuron. The shared clock generator contains a configurable ring oscillator and a clock divider. The length of the ring oscillator and the divisor for the clock divider are determined during testing to obtain the desired clock frequency. The first falling edge of the clock generator’s output after an active FF_clk–en_ sets the un-gate flip-flop (FF_clk–ug_) to high, ungating the clock signal that goes into the FSM. The use of FF_clk–ug_ ensures that there is a complete low phase of the clock signal before the rising edge at the clock input of the FSM, giving sufficient setup time to the flip-flops in the FSM.

Once awoken, the neuron FSM gets executed. The FSMs are slightly different for the input core, hidden cores, and output core (Figures 2B–D). In the case of hidden neurons, the FSM, as shown in Figure 2B, enters the *Potential Update* state on receiving the positive edge of the clock. The neuron’s potential is increased or decreased by one based on the input spike’s type. Then, the neuron’s potential is compared with the preset threshold (TH) in *Check Pot.* State. The neuron contains a 9-bit adder/subtractor to increment/decrement potential and to compare the potential with the threshold. If the potential is less than the threshold, the FSM goes back to the *Start/Standby* state while resetting all the flip flops in the wake-up circuit (FF_+__1_, FF_–__1_, FF_clk–en_, and FF_clk–ug_; find them in Figures 3A,B). Otherwise, it resets the neuron’s potential to zero and also FF_+__1_ and FF_–__1_ in the *Potential Reset* state, allowing for receiving the next spike (Figure 3C). The FSM then enters the *Spk Req* state, asserts the firing request (Req_i_) and waits for the acknowledgment (Ack_i_) from the arbiter in the synapse block. While waiting for Ack_i_, if the FSM receives a new spike it enters another state, *Potential update 2*, where the neuron’s potential is calculated. Once Ack_i_ from the arbiter is received, the neuron’s FSM goes back to the *Start/Standby* state after resetting the flip-flops (FF_+__1_, FF_–1_, FF_clk–en_, and FF_clk–ug_) in the asynchronous wake-up circuits. This cuts off the clock and power to the neuron.

The operation of input and output neurons are slightly different. The input neuron’s FSM is depicted in Figure 2C. On receiving a spike, the FSM directly enters the *Spk Req* state, asserts a firing request (Req_i_) and waits for an acknowledgment (Ack_i_) from the arbiter in the synapse block. On receiving Ack_i_ from the arbiter, the FSM resets FF_+__1_, FF_–1_, FF_clk–en_, FF_clk–ug_ and goes back to the *Start/Standby* state. Again, in this state, the clock and power to the neuron are gated. The output neuron’s FSM is depicted in Figure 2D. Upon receiving a spike, the FSM enters the *Potential Update* state, then in the next state, it resets FF_+__1_, FF_–1_, FF_clk–en_, FF_clk–ug_ and then goes back to the *Start/Standby* state. The output neuron does not generate any spikes and only keeps track of the potential. The neuron with the highest potential determines the classification result.

**FIGURE 4:**
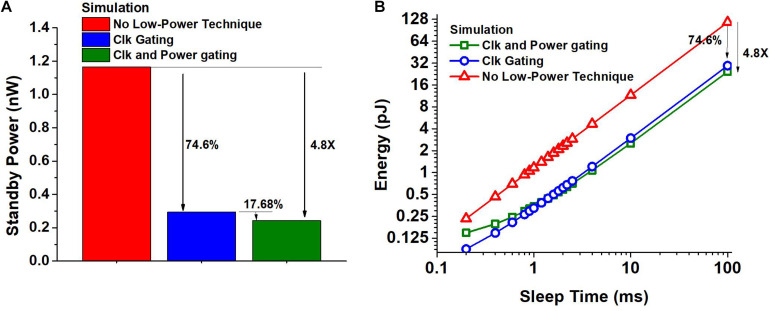
**(A)** Impact of spike-driven clock gating and a combination of clock and power-gating on the standby power consumption of a hidden neuron obtained using SPICE simulation. **(B)** Energy consumption of the hidden neuron obtained using SPICE simulation as a function of sleep time between two spikes, when the clock is free running and when clock gating and power gating are used.

This spike-based event-driven operation enables large power reduction and energy savings. First, if the input has no activity, which is common for always-on applications, the proposed neuron architecture can enjoy a very long sleep time. The hidden neuron without spike-event-driven power management would consume 1.16 nW as shown in Figure 4A. The proposed clock-generation/-gating enables 74.6% power savings and the zigzag power gating provides an additional 17.68%, resulting in an overall power reduction of 4.8X when the circuit is not processing any spikes.

If the input has non-zero activity, the proposed neuron will experience shorter sleep time but it still saves a considerable amount of energy. For the targeted benchmarks, the shortest idle time between two spikes per neuron is estimated to be around 4 ms at the maximum input rate. Figure 4B shows the energy consumption of the hidden neuron as a function of sleep time obtained using SPICE simulation. The energy consumed includes the overhead of transitioning in to and out of the power down state and the energy consumed during sleep. We consider the hidden neurons with no low power technique used, with only clock gating used, and with both clock and power gating used. We can observe that the neuron with clock and power gating can save energy consumption by 4.35X for 4 ms sleep time. Also, if the sleep time of the neuron is greater than 1.3 ms, we stand to gain due to the proposed fine-grained clock and power gating. The shortest idle time between two spikes would be much smaller for SNN accelerators that target high throughput, making it challenging to obtain any benefit from fine-grained power gating.

### Synapse Block

**FIGURE 5:**
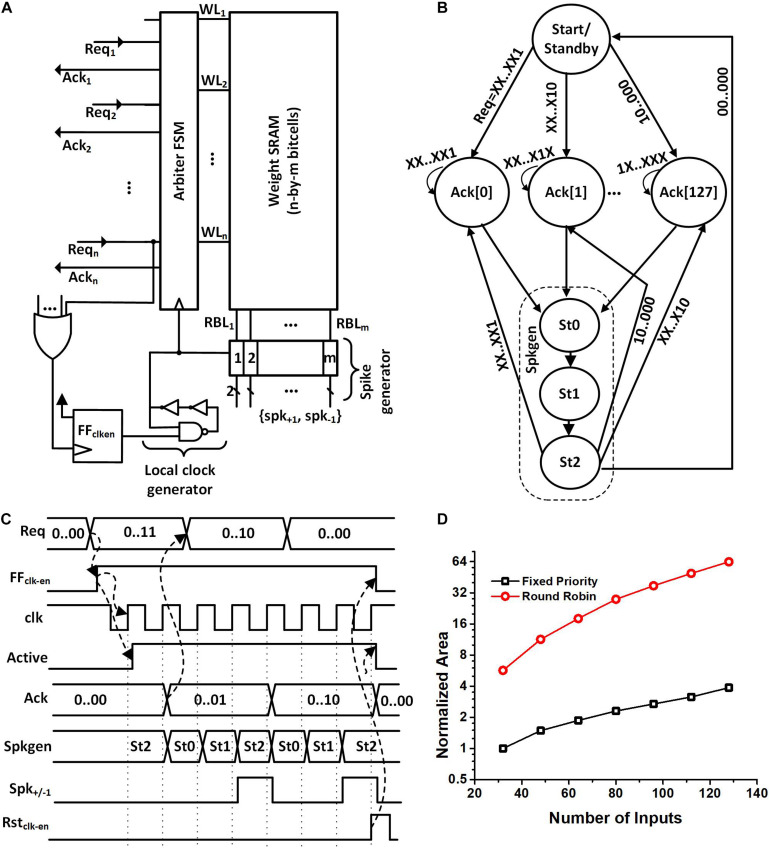
**(A)** Proposed synapse block architecture. **(B)** Arbiter FSM showing the fixed priority and Spkgen sub-FSM. **(C)** Waveforms showing the operation of the synapse block when neuron 1 and 2 generate a request. Acknowledgment is given to neuron 1 because of higher priority. Spkgen sub-FSM executes while the acknowledgment is high. **(D)** Normalized area comparison between round-robin arbiter and fixed priority arbiter for a different number of inputs. The normalized area is obtained by dividing the cell area with the area of the fixed priority arbiter with 32 inputs.

The synapse block was also designed based on the event-driven architecture. Figure 5A shows its microarchitecture. The synapse block has an arbiter FSM, an SRAM array, spike generators, and its own clock generator. A request signal (Req_i_) from the neurons within the same neurosynaptic core starts the local clock generator of the synapse block, which makes the arbiter FSM get executed. In case multiple neurons assert Req_i_, the arbiter handles the requests, i.e., grants access to the single-port weight SRAM based on a fixed priority. To serve n-th neuron’s request, the arbiter asserts the n-th wordline (WL_n_) and loads the binary weights on the read-bitlines (RBLs) whose values are captured by the flip flops. Each row of the SRAM contains 128 binary weights which are equal to the number of neurons in the neurosynaptic core. This means all the weights needed to serve a neuron’s request are obtained in a single access. The spike generator uses these weight values to generate 128 positive or negative spikes to the neuron in the next layer. It is to be noted that the spike generator is connected to the neurons in the next neurosynaptic by wires only. The arbitration among the neurons also has the effect of managing access to these wires by allowing only one spike per wire at once. Therefore, we avoid the loss of information due to the collision between two (post-synaptic) spikes traveling to a single neuron at the same time.

When the local clock generator is enabled, the arbiter FSM gets executed (Figure 5B). The FSM starts in the *Start/Standby* state and when the positive edge of the clock arrives the FSM moves to one of the *Ack[i]* states. The exact *Ack[i]* state is determined based on the indices of the neurons making the request. The neurons with a smaller index have a higher priority.

The waveforms in Figure 5C show an exemplary operation of the circuit when neuron 1 and neuron 2 generate a request at the same time. We can see from the figure that once the requests are generated, the FF_clk–en_ flip-flop is set. This turns on the local clock generator and disables power gating. Acknowledgment (Ack_1_) is provided to neuron 1 because it has a higher priority determined in design time. The same acknowledgment signal acts as the read WL_n_ for the SRAM.

The arbiter then starts executing the spike generation sub-FSM (*Spkgen*). The *Spkgen* waveform in Figure 5C shows the state of the sub-FSM. When Spkgen is in the state *St1*, weight values are captured in flip-flops and when Spkgen enters state *St2*, 128 positive or negative spikes (spk_+__/__–__1_) are generated for all the neurons in the next layer based on the weight values. The arbiter acknowledges back to neuron 1 by asserting Ack_1_ while the spike generator goes through the states *St0*, *St1*, and *St2*. Ack_1_ stays high until the request from the neuron is high or the spike generation completes, whichever is later. If there are any outstanding Req_i_, the arbiter FSM continues to serve, otherwise, the clock and power are disabled.

We chose the fixed priority arbiter instead of a round-robin one as the area saving is about 17X for 128 inputs. Figure 5D shows the area of the round-robin arbiter and fixed priority relative to a fixed priority arbiter with 32 inputs. We can see from Figure 5D that the area required for a round-robin arbiter is superlinear as a function of the input size while the area for a fixed priority arbiter increases approximately linearly with the number of inputs.

The fixed priority scheme, however, could cause the neuron with the lowest priority to starve, i.e., its requests may not be served if the arbiter is busy serving the requests of the neurons with higher priority. We can address the problem of starvation by increasing the bandwidth of the SRAM or reducing the requests that neurons make. In our design process, we ensure the fixed priority arbiter starves no neuron. We improved the bandwidth of SRAM using supply boosting which is discussed in section “On-Chip SRAM.” We chose the thresholds of the neurons and the clock frequencies of the neuron and synapse blocks so that spikes are not missed while the neurons are waiting for acknowledgment from the arbiter.

**FIGURE 6:**
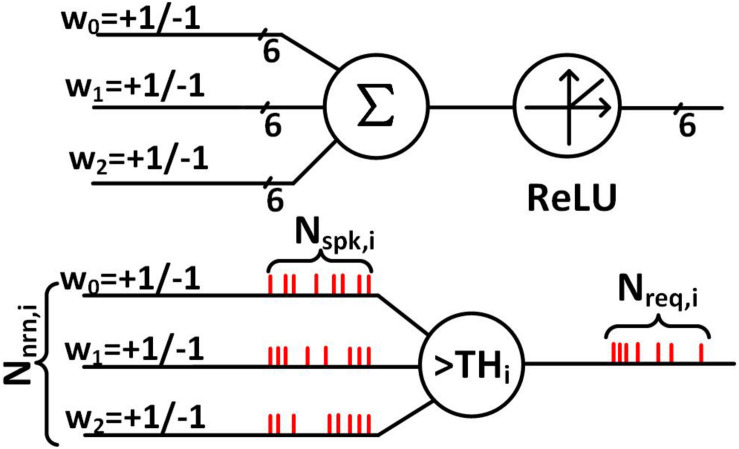
Binary coding in a BNN and spike-rate coding in an SNN.

The process to determine those key design parameters is as follows. As shown at the bottom of Figure 6, we have considered a case where a neuron receives spikes from *N*_nrn,i_ neurons and produce spikes, where we can formulate the number of requests in the *i*-th layer (*N*_req, i_), which is:

(1) $$N_{r\,e\,q,i}=\frac{N_{s\,p\,k,i}\times N_{n\,r\,n,i}}{T\,H_{i}},$$

where *TH*_i_ is the threshold of the neurons, *N*_spk, i_ is the number of incoming spikes in a particular time period (called a frame) and per neuron, *N*_nrn, i_ is the number of neurons, all in the *i*-th neurosynaptic core. On the other hand, the number of requests that the arbiter in the *i-*th layer can serve (*N*_serve, i_) can be formulated as:

(2) $$N_{s\,e\,r\,v\,e,i}=\frac{f_{c\,l\,k,a}\times T_{f\,r\,a\,m\,e}}{N_{c\,y\,c,a}}$$

where *N*_cyc, a_ is the number of cycles that the arbiter consumes to serve one request, *T*_frame_ is the frame size, *f*_clk, a_ is the arbiter’s clock frequency.

**FIGURE 7:**
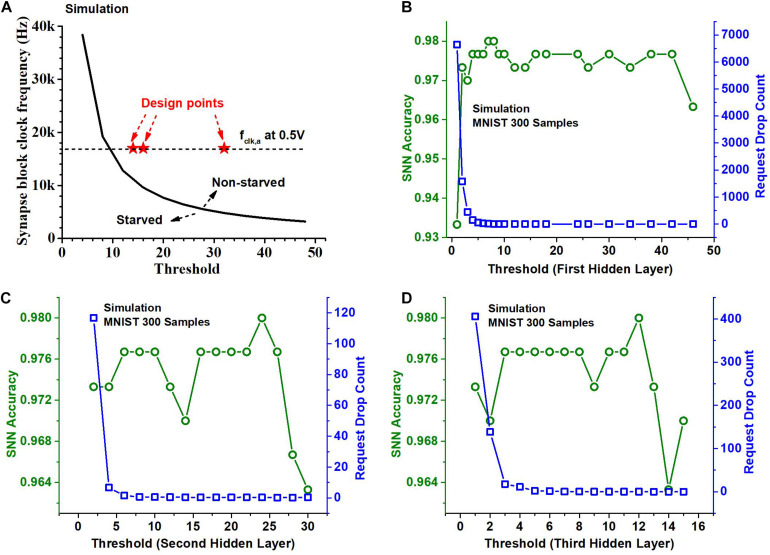
**(A)** Threshold and clock frequency optimization for no starvation during operation. Neurons are not starved when the arbiter clock frequency and the threshold for the layer are high enough. **(B)** SNN accuracy on 300 samples of the MNIST dataset and the total number of neuron requests dropped (request drop count) per sample when the threshold value for the 1st hidden layer is varied. **(C)** SNN accuracy and Request drop count when the threshold value for the 2nd hidden layer is varied. **(D)** SNN accuracy and Request drop count when the threshold for the 3rd hidden layer is varied.

If *N*_req, i_ (Eq. 1) exceeds *N*_serve, i_ (Eq. 2), starvation occurs. When starvation occurs, incoming spikes can get dropped as the arbiter is not fast enough to serve all the requests. We ensure by design no spike is dropped, i.e., by making *N*_req,i_ not exceed *N*_serve,i_. This is done by increasing *TH*_i_ or increasing *f*_clk, a_. The former, however, can incur a degradation in the accuracy. This is because the increase of TH_i_ would reduce the number of output spikes generated in the *i*-th layer. It has the same effect as reducing the precision of the activations in a binary-weight neural network that has a similar network structure. On the other hand, increasing *f*_clk,a_ increases the power consumption of the synapse block. Therefore, we swept *TH*_i_ and *f*_clk, a_ values to find optimal operating points for the chip. Figure 7A shows a curve obtained using RTL simulation with 1000 MNIST test samples (LeCun et al., 1998) and 8-bit activations. The curve separates the regions where the neurons starve and where they do not. We choose design points so that the average number of spikes per neuron is roughly the same for each of the hidden layers and hence the curve that separates the starvation and non-starvation region is the same for all of them. The design point, i.e., the neuron threshold and the synapse block clock frequency for each of the hidden layers is indicated as a red star in Figure 7A. The threshold values we chose for the hidden layers for the MNIST dataset are (32, 16, and 14) and the threshold values we chose for the KWS datasets with 6-bit activations are (28, 18, and 10). The threshold values are not very different for the two kinds of datasets despite the difference in the desired spiking rate because of the dependency on weight and input data.

Indeed, the threshold value affects the number of spikes generated in a layer and this affects the inference accuracy. Recall that the activations are spike-rate-coded multi-bit values and the reduction of the number of spikes leads to fewer bits. We can observe the impact of the choice of threshold values for different hidden layers on the accuracy of the SNN classifier in Figures 7B–D. It shows through a Python simulation the accuracy obtained on 300 test samples of the MNIST dataset. The Python simulation models the neuron and arbiter and uses a set of time series vectors as input spike train whose entries are either 1,−1, or 0 indicating the presence and the sign of the spike. We chose the time resolution so that the results mimic the RTL simulation. For Figure 7B, we varied the threshold for the first hidden layer while the thresholds of the second and third hidden layers are chosen to be 16 and 8. For Figure 7C we varied the threshold of the second hidden layer and kept the threshold of the first hidden layer to be 24 and that of the third hidden layer to be 8. For Figure 7D we varied the threshold of the third hidden layer and kept the threshold of the first hidden layer to be 24 and that of the second hidden layer to be 16. From Figure 7B we can observe that the accuracy of the classifier is worse for the small (roughly < 5) and the large threshold values (roughly > 40). Figure 7B also shows the total number of neuron requests dropped across layers as a function of the threshold of the first hidden layer. It indicates that if the threshold is too small, too many spikes are produced, causing starvation, which leads to too many neuron requests being dropped, resulting in a deterioration in the accuracy. Figures 7C,D show a similar trend when the threshold for the second hidden layer and the third hidden layer is varied. But we can also observe that the impact of the threshold of the second and third hidden layer on accuracy is relatively small if a proper threshold is chosen for the first hidden layer. This is because the number of spikes and hence the number of neuron requests are large in the first hidden layer. The threshold of the first hidden layer determines the number of requests dropped in the first hidden layer which is also a large portion of the total number of requests dropped.

#### On-Chip SRAM

The chip has 65.25 kb of SRAM and so it was important to minimize SRAM leakage power dissipation. We designed the SRAM based on the circuit described by Cerqueira et al. (2019) for ultra-low-power operation. High threshold voltage (HVT) transistors with three times minimum length were used for the bitcell to reduce leakage. The buffer in the peripheral circuits employed zig-zag power gating with cut-off transistors separate for each row, ensuring fast wake-up.

**FIGURE 8:**
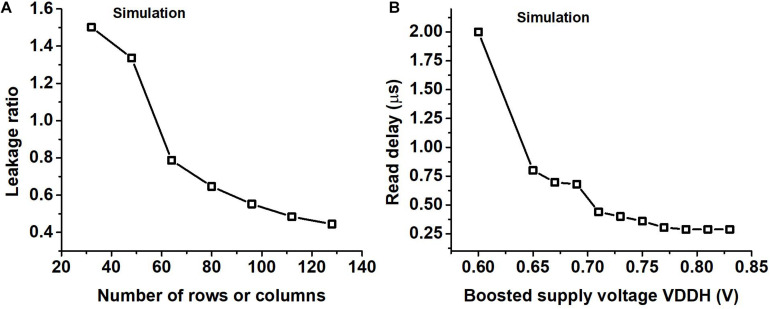
**(A)** The ratio of the leakage of the peripheral circuits to that of the bitcells (leakage ratio) for different SRAM sizes where the number of rows is the same as the number of columns obtained using SPICE simulation. **(B)** Read delay of the SRAM for different boosted supply voltages (VDDH) obtained using SPICE simulation.

We chose to have all the weights for a layer in a single SRAM macro size instead of smaller banks. Figure 8A shows the ratio of the leakage of the peripheral circuits to the leakage of the bit cells as a function of the number of rows obtained using SPICE simulation. From Figure 8A we can see that because of this the leakage of peripheral circuits would get amortized among more bitcells, helping us in the overall objective of reducing the leakage.

We use supply voltage boosting during a read operation to speed up the charge or discharge of the read bitline. The delay in the read operation arises mostly from charging the read WL_n_ and charging or discharging the read bit line. Supply voltage boosting was needed to improve the speed of read operation which took a hit due to the use of a single SRAM macro for storing all the weights in a layer. We performed a transistor-level SPICE simulation to observe the read delay of SRAM for different values of the boosted supply voltage and the results are shown in Figure 8B. In Figure 8B we can observe that on increasing the boosted supply voltage (VDDH) we will eventually be limited by the time taken to charge the WL_n_. We operate our circuit so that read delay is not the critical path in the design by choosing a high enough VDDH, which is roughly 0.8 V if the regular VDD is set to 0.52 V.

### Experiment Setup

#### Chip Prototype

**FIGURE 9:**
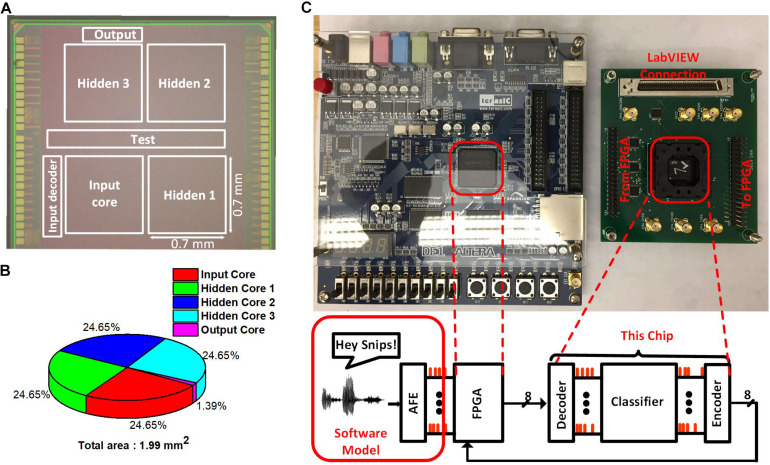
**(A)** Die photo of the SNN Classifier with the Neurosynaptic Cores along with the Test Circuits. **(B)** Area break-down of the SNN Classifier. **(C)** Test chip with its connection to the FPGA board and LabVIEW. FPGA interface is used for sending the inputs to the chip and reading out the potential of the output neurons. LabVIEW is used for configuring the thresholds and write to the SRAMs.

We prototyped the test chip in a 65 nm LP CMOS process. Figure 9A shows the die photo with the boundaries of different cores marked. The input and the hidden cores have the dimensions 0.7 mm × 0.7 mm. The output neurons take an area of 0.0276 mm^2^. Each of the hidden cores is logically equivalent but have a different layout to simplify the routing. The total core area is around 1.99 mm^2^. The area breakdown of the chip can be seen from the pie chart in Figure 9B. The chip also contains the input decoder and the output encoder for reducing the number of I/O the chip would require. Those decoder and encoder convert the spike I/Os to the binary address in AER, reducing the spike I/O pin count from 512 to 9. Also, the chip contains a scan chain to configure the thresholds of the neurons in different neurosynaptic cores, set the clock frequency of the neuron and synapse blocks and write the weights into the SRAMs.

#### Input Preparation

We envisioned the SNN to interface directly with a spike-generating feature-extraction front end such as the ones discussed in Yang et al. (2015, 2019, 2021a). For our experiments, we used the software model (Yang et al., 2019) to generate features for the KWS task. The software model makes use of post-layout Spectre simulations for tuning its parameters and has been validated using chip measurements. In the analog front end, the spikes are generated when the voltage on a capacitor exceeds a certain threshold (Th_analog_). The finite bandwidth of the comparator and Th_analog_ together control the spike frequency. We do not alter the value used for Th_analog_ across the HeySnips and the GSCD datasets.

The software model generates features of size 16. Each dimension of the feature captures the energy at a central frequency in the form of the number of spikes that are generated by the analog front end in a certain time period. We call this time period as frame size in case of audio input. The central frequencies of the 16 channels are geometrically scaled from about 100–5 kHz. We configure the front end so that the number of spikes can be represented by 6-bits, i.e., each element in the feature has 6-bit precision. We set a frame size *T*_frame_ of 80 ms with no overlap between successive frames, based on the length of the audio clip of the datasets and the dimension of the input layer that the chip supports. In GSCD and HeySnips datasets, each keyword audio sample is roughly 1s. We put together the feature vector of the current frame along with the feature vectors of the past 15 frames to obtain a vector of size 256 that contains the number of spikes associated with each input neuron. We evenly spread out the spikes for each input neuron within a time period equal to the frame size. The FPGA then sends these spikes to the input decoder inside the SNN chip in the form of AER.

In the case of the MNIST grayscale dataset, we downsample the image size to 16 × 16 by utilizing 2 × 2 max-pooling so that we can match the image with the size of the input layer of the chip. For each input sample, we generate a set of time series vectors (spike trains) for the input layer of the chip in a time duration equal to the frame size, which is chosen based on the latency of the chip.

#### Training

We train a binary neural network (BNN) model that uses binary weights (+1, −1), has no bias and 6-bit ReLU activation (Cao et al., 2014) for the KWS task. The network structure is equivalent to the SNN model we deploy. The BNN provides the weights for the SNN model. The 6-bit activations in the BNN are encoded for the SNN using spike-rate, e.g., 010000_(2)_ is mapped to 16 spikes/frame. We set the threshold of the neurons in each layer such that each neuron generates at most 63 spikes/frame (i.e., N_spk,i_ in Figure 6 is less than 63), which matches the 6-bit activation of the BNN model. This is possible because in the SNN model, as spikes pass through the neurons in a layer, the number of spikes scales roughly by the ratio of the threshold. We can easily change the activation precision after deployment for different models by configuring the thresholds. For example, we use 8-bit activation with the *T*_frame_ of 0.5 s for the MNIST grayscale.

#### Inference Testing

Altera DE1 board containing a Cyclone II FPGA chip is used to interface with the input decoder and the output encoder in the SNN chip as shown in Figure 9C. The SNN chip along with the FPGA is globally synchronous but locally asynchronous. The global clock comes from the FPGA and is used to send new inputs and read out the potential of output neurons at regular intervals. The input decoder and the output encoder are synchronized to the global clock but are asynchronous to the clock of the input core and the output core. All the neuron and synapse blocks within the SNN chip are asynchronous to each other. LabView is used to configure the scan-chain and write weights into the SRAMs in the neurosynaptic cores.

The FPGA board reads out the input data from its memory. It sends an 8-bit AER code to the input decoder identifying the neuron which is supposed to receive a spike and another signal identifying whether the spike is an incrementing spike or decrementing spike. The input decoder then sends a pulse to the appropriate neuron. Spikes that arrive at the input of the hidden layer arrive at all the neurons simultaneously. There is separate hardware for each neuron and hence they can process spikes simultaneously and compete for access to the SRAM. After an interval greater than or equal to the latency of the chip, the FPGA deactivates the output core’s clock so that no more spikes are processed. It then enables the output encoder to read out the potential of the output neurons in a serial fashion. The SNN chip is not pipelined, so at the end of the readout, the FPGA resets the potential in all the neurons and sends in the next set of spikes to the input decoder.

For our experiments, we define the latency of the SNN chip as the time needed to process enough spikes to achieve the desired accuracy. If the latency of the chip is less than or equal to the frame size, we can achieve real-time operation. We can stream a new input to the classifier at the end of each frame, which is typically done in audio processing systems. The time period we allow the chip to process is equal to the frame size. The frame sizes are large enough to process most of the spikes and not hurt the accuracy of the task.

## Results

**FIGURE 10:**
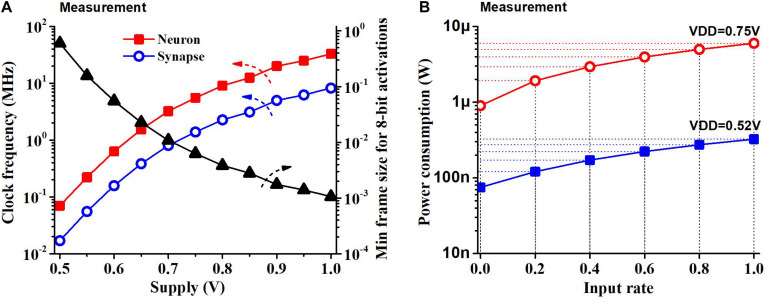
**(A)** Clock frequency measurement and min frame length for 8-bit activations as a function of the supply voltage. Frame size is constrained by the latency of the SNN classifier (i.e., longer latency increases the minimum frame size). **(B)** Power consumption of the chip as a function of the input rate at two supply voltages. Power consumption increases linearly with the input rate.

Most of the results are based on a supply voltage of 0.52 V and the clock frequency of the neuron block of 70 kHz and that of the synapse block of 17 kHz, while the chip can operate at other supply voltages and achieve different frequencies. Figure 10A shows the measurement results of the neuron block frequency, synapse block frequency and latency of the chip (minimum frame size) at different supply voltages when 8-bit activations are used. An off-chip instrument (NI LabView) was used to measure the clock frequency. The latency of the chip was measured by comparing the potential of the output neurons with the results from RTL simulation for 50 samples of the MNIST test set with 8-bit activations. The minimum frame size we can use to operate the chip with 8-bit activations reduces with a supply voltage as the speed of the circuit increases.

We measured the power consumption of the chip during standby and when continuously running (100% input rate) KWS datasets like GSCD or HeySnips. The power consumption of the chip would scale based on the amount of activity at the input. Figure 10B shows what the SNN chip power consumption would be at different input rates. We obtain the maximum switching power by subtracting the standby power from the power consumption at a 100% input rate. We obtain the power consumption at an intermediate input rate by scaling the switching power and summing it up with the standby power. The SNN chip dissipates a power of 75 nW when there is no input and power of 220 nW when running a KWS dataset at a supply of 0.52 V.

**FIGURE 11:**
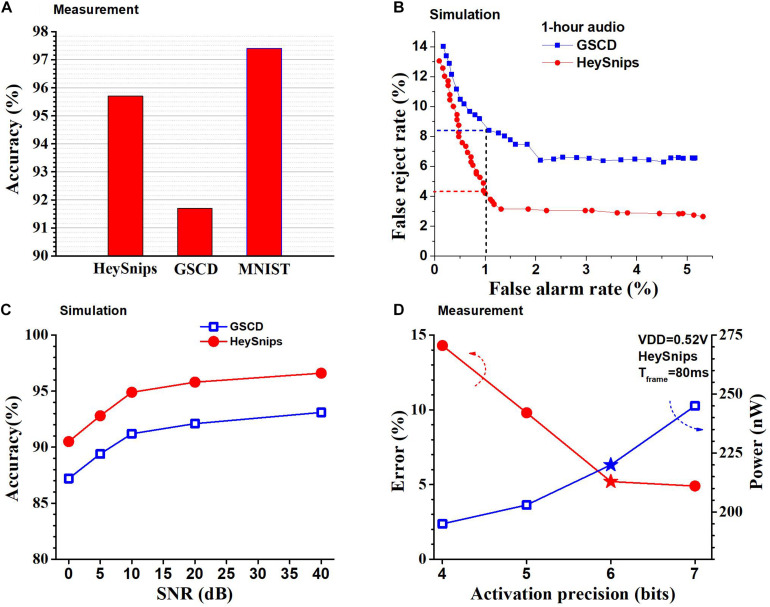
**(A)** Accuracies of the SNN chip measured across multiple benchmarks. **(B)** ROC curves from KWS benchmarks obtained using RTL simulation. **(C)** Accuracy of the SNN chip on KWS datasets across 0–40 dB SNR levels obtained using RTL simulation. **(D)** Measured power consumption of chip and error for the HeySnips dataset as a function of activation precision. Stars denote the operating point used for comparison with other works.

We *physically measured* the accuracy of the chip and we can see the accuracy of the chip across the different classification tasks in Figure 11A. We read out the output neurons’ potential to the FPGA and pick the index of the neuron with the highest potential as the predicted class. In GSCD, the SNN can recognize four keywords (“yes,” “stop,” “right,” and “off,” arbitrarily chosen) and fillers with an accuracy of 91.8%. The SNN architecture we use is 256-128-128-128-5 and configure the thresholds to be (1, 28, 18, 10) where 1 is the threshold for the input layer (fixed) and the rest are for the hidden layers. For the HeySnips dataset, the chip can recognize one keyword (“Hey Snips”) and fillers with an accuracy of 95.8%. For the MNIST grayscale dataset, the trained SNN structure is 256-128-128-128-10 with the thresholds of (1, 32, 16, 14) and it gives an accuracy of 97.6%.

Figure 11B shows the receiver operating characteristic (ROC) curve for GSCD and HeySnips. It shows the false reject rate (FRR) as a function of the false alarm rate (FAR) for 1-h-long audio obtained by concatenating the test set samples and running an RTL simulation. FAR indicates the number of false positives while FRR indicates the number of false negatives. We obtained the ROC by calculating the softmax of the output neurons’ potential and varying the discriminating threshold. If the softmax value of the keyword class is greater than the discriminating threshold then the prediction is a keyword otherwise it is a non-keyword. If the discriminating threshold is large (close to 1) most of the audio frames will be classified as non-keyword which will increase the number of false negatives (FRR). If the discriminating threshold is small (close to 0) then most of the audio frames will be classified as keyword thereby increasing the number of false positives (FAR). In the case of GSCD, we take the average of the pairs (FAR and FRR) we obtain for each keyword at a certain discriminating threshold. In addition, we ran an RTL simulation to obtain the accuracy of the chip in the presence of noise by mixing the speech audio with white noise at various SNRs. We adopted noise-dependent training for this experiment (Yang et al., 2019), i.e., we use the same SNR for both train data and test data. The SNN classifier chip achieves reasonably high accuracy across 0 to 40 dB SNR levels for both GSCD and HeySnips datasets as shown in Figure 11C. The configurability of the thresholds of different layers in the SNN classifier architecture allows us to change the data precision after deployment. Recall that changing the threshold in the hidden layers of the SNN has the same effect as changing the precision of the activations in a deep neural network with the same network structure and that activation is spike rate coded in our SNN. This can be used to trade-off accuracy for power savings. Figure 11D shows the measured accuracy and the power consumed by the SNN chip when the precision of the activations is varied for the HeySnips dataset. At higher activation precision the error is lower, but the power consumption is higher and at lower activation precision the error is higher, but the power consumption is lower. We chose a precision of 6-bit which is a good compromise between the power consumption and the accuracy.

**FIGURE 12:**
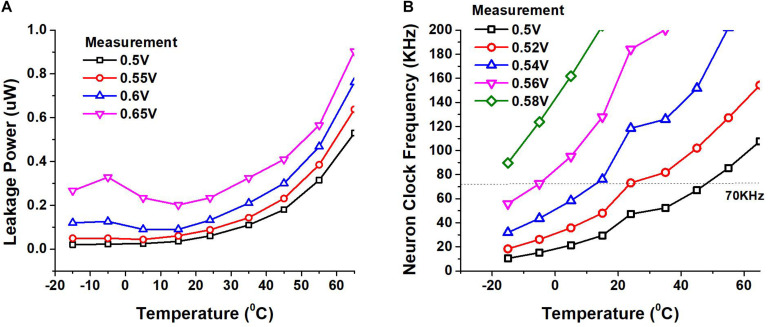
**(A)** Leakage power of the chip measured as a function of temperature at different supply voltages. **(B)** Variation of neuron clock frequency measured as temperature and supply voltage vary.

We also measured the impact of temperature on the leakage power dissipation and the speed of our circuits. We placed our SNN chip and other testing hardware in a temperature chamber for our measurements. Figure 12A shows the leakage power of the circuit while Figure 12B shows the clock frequency of the circuit at different supplies as the temperature is varied. The margin we provide to the length of the ring oscillator helps us avoid timing failure due to temperature, supply and process variation to a certain extent. While our design does not have a mechanism to dynamically tune the supply voltage or frequency, it is beneficial to operate the circuit at a lower supply when the temperature is high and at a higher supply when the temperature is low, to obtain the needed performance while keeping the power consumption low.

**FIGURE 13:**
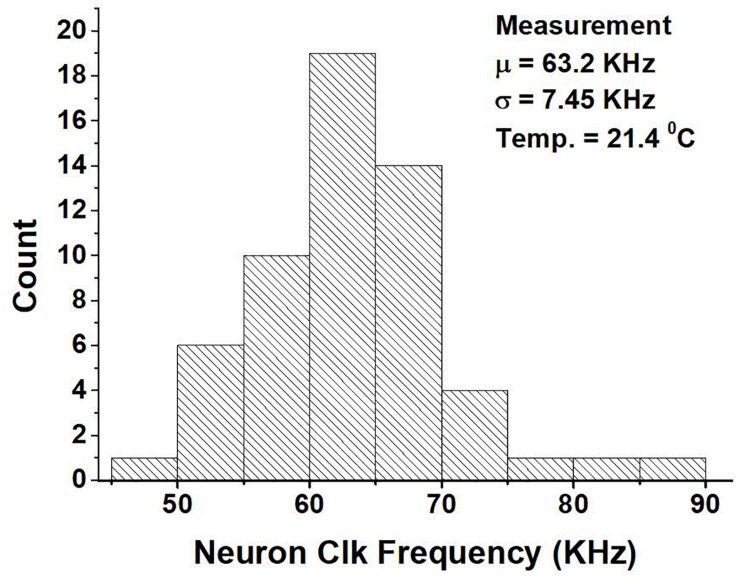
Measurement of the neuron clock frequency of around 50 neurosynaptic cores across 10 chips at a supply of 0.52 V.

On the other hand, Figure 13 shows the variation in the neuron clock frequency among approximately 50 cores across 10 chips at a supply of 0.52 V. From the figure we can see that the mean is 63.2 KHz and the standard deviation is 7.45 KHz. The variation in the clock frequency is due to both the difference in the layout of the ring oscillators across cores and chip-to-chip variation. The chip-to-chip variation among the cores is not uniform. The standard deviation of the clock frequency varies from 5.6 to 9.5 KHz based on the specific core. If we use the average of the clock frequency of cores within a chip as being indicative of the chip’s performance, the chip used for comparison and reporting other measurements has a performance that is about average.

## Discussion

Prior works on SNN hardware have focused on non-always-on application (Akopyan et al., 2015; Chen et al., 2018; Davies et al., 2018), support for on-chip training (Chen et al., 2018; Davies et al., 2018; Park et al., 2019) and support for both deep learning and neuromorphic workloads (Pei et al., 2019). The absence of any prior work on SNNs for targeting always-on hardware motivated us to explore a new architecture for SNNs.

We presented a fully spike-event-driven SNN classifier for an always-on intelligent function. We employed a fine-grained clock and power-gating to take advantage of the input signal sparsity, low leakage SRAM and a fixed priority arbiter to achieve a very low standby power of 75 nW. We trained the SNN for multiple always-on functions, notably multi- and single-keyword spotting benchmarks, achieving competitive accuracies. The average power consumption of the SNN chip scales with the input activity rate. It ranges from 75 nW with no input activity and 220 nW with the maximum input activity for the KWS benchmarks.

Table 1 summarizes the comparison of our work with other recent KWS accelerators. Our design achieves 2.3–6.8X power savings compared to Shan et al. (2020) among KWS accelerators. If we scale the area of our design to 28 nm it would be 0.37 mm^2^ which is still slightly higher than Shan et al. (2020). The higher area usage of our work is possibly because it does not adopt time-sharing in neuron hardware.

**TABLE 1:** Comparisons with recent KWS hardware.

	This work	Shan et al. (2020)	Guo et al. (2019)	Giraldo and Marian (2018)
Technology (nm)	65	28	65	65
Algorithm	SNN	DSCNN	RNN	LSTM
Area (mm^2^)	1.99	0.23	6.2	1.035
VDD (V)	0.52–1	0.41	0.9–1.1	0.575
Clock frequency	70 kHz @ 0.52 V	40 kHz	75 MHz	250 kHz
Benchmark 1	GSCD (4 Keywords)	GSCD (2 Keywords)	GSCD (10 Keywords)	TIMIT (4 Keywords)
Accuracy (%)	91.8	94.6	90.2	92.0
Benchmark 2	HeySnips (1 Keyword)	GSCD (1 Keyword)	HeySnips (1 Keyword)	N/A
Accuracy (%)	95.8	98.0	91.9	N/A
Power	75–220 nW*	510 nW**	134 μW	5 μW

*Power consumption scales with input rate; **feature extraction circuits included.

Our work does not have feature extraction circuits. They would increase the area and power when included. We can consider two feature extraction circuits (Yang et al., 2019, 2021a), as candidates for the analog front end for our chip. Yang et al. (2021a) is the improved version of Yang et al. (2019). We used the software model of the analog front end presented in Yang et al. (2019). The power consumed by the analog front end and the feature extraction circuits is 50 nW in the improved version and 380 nW in the older version.

The use of multiple supplies (VDD = 0.52 V and VDDH = 0.8 V) in our work can add some hardware and power overhead. There would be a significant increase in power consumption if we use only 0.8 V as the power supply for our chip. For example, if we assume that power consumption increases quadratically with VDD, then the power increases by 2.4X. We can consider two scenarios that can provide two different supplies and avoid a large increase in power. In one scenario, we assume an external DC-DC converter provides VDD while we can generate VDDH using a capacitor-based charge pump circuits (Kim et al., 2021). The current load of the VDDH is not high since it is used in only a small part of SRAM. Therefore, even if the charge pump efficiency is not high, the overall impact is small. In the other scenario, we assume an external DC-DC converter provides VDDH and then we can generate VDD using an on-chip digital LDO. This LDO would have a power efficiency of 65% (VDD/VDDH), which increases total chip power dissipation by 53.8%.

Table 2 summarizes the comparison of our design with other SNN hardware work (TrueNorth’s power is estimated from Cheng et al., 2017). Our design achieves over 30,000X power savings compared to Chen et al. (2018) in Table 2. Our design is optimized for ultra-low power always-on functions while others are optimized for a balance between higher throughput and energy efficiency. High-performance SNN accelerators generally assume that input will be presented at a much higher rate, therefore, the time interval between spike events would be much smaller, limiting the benefit of fine-grained clock gating. Our design achieves competitive accuracies among both KWS and SNN hardware works and contributes to a growing body of literature that supports SNNs as an attractive low-power alternative to deep learning based hardware architectures.

**TABLE 2:** Comparisons with recent SNN hardware.

	This work	Koo et al. (2020)	Park et al. (2019)	Chen et al. (2018)	TrueNorth
Technology (nm)	65	90	65	10	28
Neuron count	650	1	410*	4096	1M
Synapse count	67k	1	N/A	1M	256M
Area (mm^2^)	1.99	0.15	10.08	1.72	430
Clock frequency	70 kHz @ 0.52 V	37.5 MHz	20 MHz	105 MHz @ 0.5 V	N/A

	**MNIST classification**				

Power	305 nW	282.8 mW^†^	23.6 mW	9.42 mW**	63 mW
Accuracy (%)	97.6	92.3	97.8	97.9	97.6***
Throughput (inf/s)	2	N/A	100K	N/A	N/A
Energy per inference (nJ)	195	N/A	236	1700	N/A
Energy per SOP (pJ)	1.5	8.4 pJ/1.84 pJ^††^	N/A	3.8	26

*Input layer not included; **estimated from neuron’s power dissipation; ***estimated from Hsin-Pai Cheng et al., IEEE DATE 2017; ^†^power reported in Koo et al. (2020) based on network size and power for one neuron and synapse; ^††^energy with sequencing circuits / Energy without sequencing circuits.

## Data Availability Statement

The original contributions presented in the study are included in the article/supplementary material, further inquiries can be directed to the corresponding author.

## Author Contributions

PC and DW designed the chip with PC focussing on the synapse block. DW focussing on the neuron. SK contributed to improving the tape-out flow. JC designed the bitcell for use in the SRAM block while MY contributed to the software that simulates the analog front end. MS supervised the project and is the principal investigator. JK and SJ contributed to the technical discussions. All authors contributed to the article and approved the submitted version.

## Conflict of Interest

JK, SJ, and SK are employed by Samsung Electronics. The remaining authors declare that the research was conducted in the absence of any commercial or financial relationships that could be construed as a potential conflict of interest.
